# Design and implementation of compact dual-band conformal antenna for leadless cardiac pacemaker system

**DOI:** 10.1038/s41598-022-06904-2

**Published:** 2022-02-24

**Authors:** Deepti Sharma, Binod Kumar Kanaujia, Vikrant Kaim, Raj Mittra, Ravi Kumar Arya, Ladislau Matekovits

**Affiliations:** 1 School of Computational and Integrative Sciences, Jawaharlal Nehru University, New Delhi, 110067 India; 2 University of Central Florida, Orlando, FL 32816 USA; 3 Electrical and Computer Engineering Department, Faculty of Engineering, King Abdulaziz University, Jeddah, 21589 Saudi Arabia; 4 National Institute of Technology Delhi, New Delhi, 110040 India; 5 Department of Electronics and Telecommunications, Politecnico Di Torino, Turin, Italy; 6 Department of Measurements and Optical Electronics, Politehnica University Timisoara, 300006 Timisoara, Romania; 7 Istituto di Elettronica e di Ingegneria dell’Informazione e delle Telecomunicazioni, National Research Council, 10129 Turin, Italy; 8 Dr. B R Ambedkar National Institute of Technology, Jalandhar (Punjab), 144011 India

**Keywords:** Biomedical engineering, Electrical and electronic engineering

## Abstract

The leadless cardiac pacemaker is a pioneering device for heart patients. Its rising success requires the design of compact implantable antennas. In this paper, we describe a circularly polarized Hilbert curve inspired loop antenna. The proposed antenna works in the WMTS (Wireless Medical Telemetry Services) 1.4 GHz and ISM (Industrial, Scientific, and Medical) 2.45 GHz bands. High dielectric constant material Rogers RT/Duroid 6010 LM (${\epsilon }_{r}$=10) and fractal geometry helps to design the antenna with a small footprint of 9.1 mm^3^ (6 mm × 6 mm × 0.254 mm). The designed antenna has a conformal shape that fits inside a leadless pacemaker’s capsule is surrounded by IC models and battery, which are tightly packed in the device enclosure. Subsequently, the integrated prototype is simulated deep inside at the center of the multi-layer canonical heart model. To verify experimentally, we have put dummy electronics (IC and battery) inside the 3D printed pacemaker’s capsule and surfaced the fabricated conformal antenna around the inner curved body of the TCP (Transcatheter Pacing) capsule. Furthermore, we have tested the TCP capsule by inserting it in a ballistic gel phantom and minced pork. The measured impedance bandwidths at 1.4 GHz and 2.45 GHz are 250 MHz and 430 MHz, whereas measured gains are − 33.2 dBi, and − 28.5 dBi, respectively.

## Introduction

Pacemakers are essential lifesaving implantable devices for Bradycardia (slow heart rate disease) patients. The slower heart rate diseases: Syncope, hypertrophic cardiomyopathy, and heart tissue damage are the reason for cardiac arrest. More than 5 million individuals in the United States and 25 million globally are affected by cardiac arrest, which caused existence of more than 3 million pacemaker implants worldwide^1^. Since the world's first successful pacemaker implant, dating back to, 1958 conventional pacemakers have evolved rapidly^2^.

The subsequent six decades have witnessed the most significant developments in research areas of size reduction, multi-chamber pacing, device programming, battery life expectancy, rate-responsive behavior, and remote monitoring of the device^3^. Although conventional pacemakers significantly reduced the mortality rate, the infection issues remained unresolved. The subcutaneous pocket (in which the pacemaker implants) causes skin erosion and hematomas; and transvenous leads (inserted in the heart for pacing) causes pneumothorax, obstruction of central vein, tricuspid valve failure, and recurrent infections^4–7^. Therefore, to overcome the problems associated with lead and pocket, the leadless pacemaker was considered 50 years ago^3^. In 1970, during a preclinical study, an iatrogenic heart block was paced for more than two months using canine. In 1991, again in another preclinical trial, this approach was tested but could not get complete success due to the lack of device miniaturization techniques^8^. The successful implementation of a self-contained leadless pacemaker became a reality in 2013^9^ because of improvements made (after 1991) in several areas, including catheter-based delivery systems, low power electronics, miniaturized energy sources and innovative packaging capabilities.

**Figure 1:**
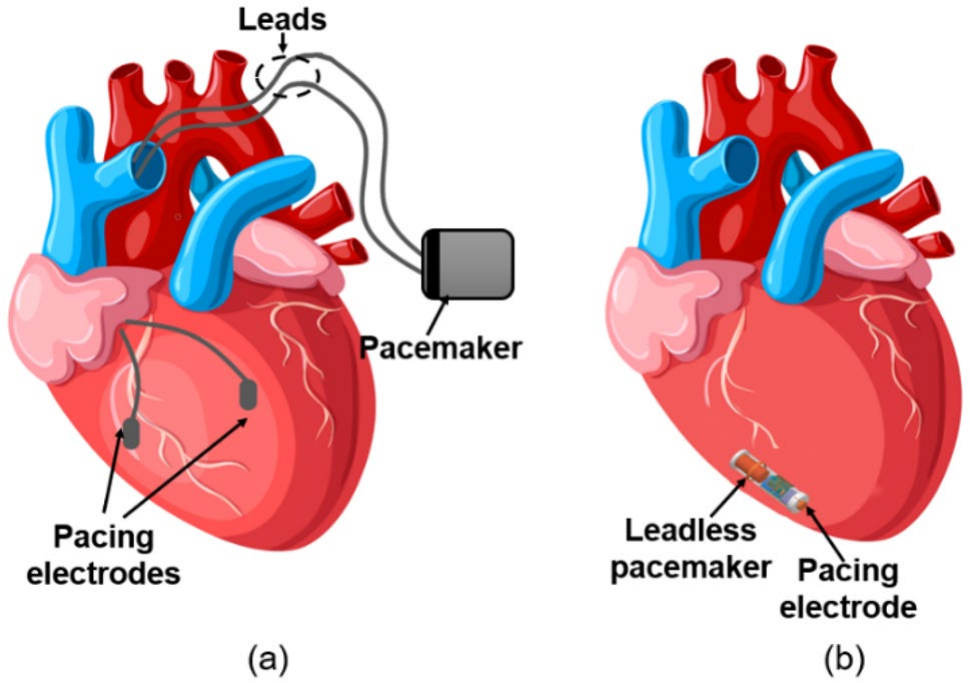
Schematic of the conventional pacemaker vs leadless cardiac Transcatheter Pacing (TCP) system (**a**) conventional pacemaker with pacing leads (**b**) leadless cardiac TCP implanted inside human heart with pacing electrode at the tip (human heart figure is adapted from^12^.

The conventional pacemaker is implanted under the skin of the chest. The pacing leads come out of the pacemaker and always placed so that it runs through a large vein in the chest leading directly to the heart. The electrode on the end of a lead touches the heart’s chamber to provide pacing, as shown in the Fig. 1. The volume of the conventional pacemaker is 960 mm^3^ (40 $\times$ 40 $\times$ 0.6) as per^10^. Whereas, the leadless cardiac pacemaker is a single component capsule-like device with volume of 544.8 mm^3^ (π $\times$ 6.67 $\times$ 26) that comprises a pulse generator and pacing electrodes in a single unit, eliminates the leads, device pocket, and intrasystem connections^9^. The leadless cardiac pacemaker implants in Right Ventricle (RV) of the heart as shown in Fig. 1. Currently, St. Jude Medical’s Nanostim^11^ and Medtronic’s Micra Transcatheter Pacing (TCP).

System^13^ are two variants of self-contained pacemakers available for implantation. Despite the several advantages of leadless TCPs over conventional pacemakers: the single-chamber pacing, battery longevity, and challenging device retrieval are limitations.

**Figure 2:**
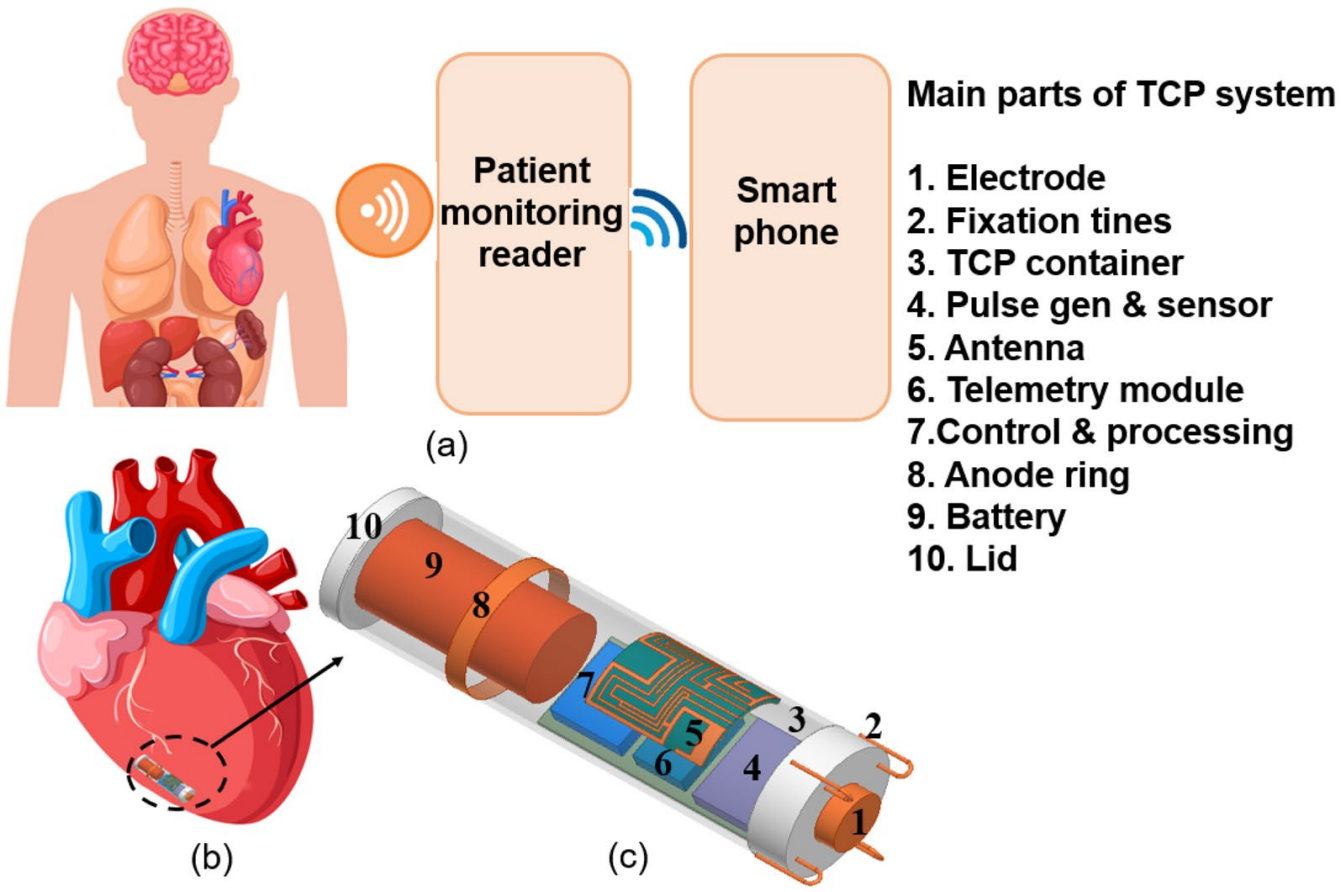
Schematic of the leadless cardiac Transcatheter Pacing (TCP) system (**a**) wireless monitoring of the implanted TCP with patient monitoring reader and data transfer to smart phone (**b**) leadless cardiac TCP implanted inside human heart. (human body figure is adapted from^12^).

Presently, biotelemetry is one of the most significant characteristics regarding active Implantable Medical Devices (IMDs), such as endoscopy capsules^14^, retinal implants^15^, neural implants^16^ and pacemakers^17,18^ which enable effective communication between the antenna of IMD and the antenna of the external patient monitoring device. Implantable antennas proposed for the biotelemetry of conventional pacemakers were presented in^10,17,19^. The implantable antenna designed in ^10,17^ considered the pacemaker’s device (dimension and material) to optimize the antenna, but in^19^ antenna was designed inside the skin phantom. similarly, the antennas for the leadless pacemaker were presented in^18,20–23^. The antenna proposed in^21^ was designed inside the heart muscle only. It is very important to design and optimize the antenna inside the device because the antenna, designed without considering the device structure, detune when placed into the device. In Medtronic Micra’s TCP system, data is transferred wirelessly from the TCP system to the healthcare professional using “my care link”^24^ patient monitoring device as shown in Fig. 2. Along with the data transfer, some advanced implantable devices also perform energy harvesting and control signaling^25^. Also, if a multiband antenna is used only for data transfer, it can offer flexible operation at different data rates^25^. So, the recent development in leadless cardiac pacemakers needs a compact and multiband antenna, which can effectively communicate to the external device. In^18^, a 400 MHz MICS band conformal antenna with a diameter of 9.46 mm was proposed. The gain of this antenna was − 32 dBi. A 14.28 $\times$ 17.28 $\times$ 0.289 mm^3^ conformal patch antenna wrapped around the body of a dummy cylinder was reported in^20^. This antenna was working in the single ISM 2.45 GHz band with a gain of − 35 dBi. A flat circular planar spiral antenna, which was working in MICS 400 MHz band was reported in^21^ for leadless pacemaker applications. Another flat compact multiband spiral antenna with dimensions of 7 $\times$ 6.5 $\times$ 0.377 mm^3^ was proposed in^22^. But the antennas designed in^18,21^ and ^22^ are quite large for leadless pacemaker’s applications because the commercially available leadless pacemakers such as Nanostim’s LCP has a diameter of only 5.99 mm with a length of 42 mm and Micra’s TCP has a diameter of 6.7 mm with a length of 25.9^3^. Recently, a flat 3 $\times$ 4 $\times$ 0.5 mm^3^ ultra-miniaturized meandered antenna compatible with TCP’s capsule; was reported^23^. This antenna was working in the ISM 2.45 GHz band, and simulated gain and bandwidth of this antenna was − 25.9 dBi and 21.8%, respectively. Although antennas designed in^20^ and^23^ are compatible for leadless pacemaker’s application, they exhibit single-band operation. The mobility of the patient with the implanted medical devices is also a key factor. Therefore, to set up a strong communication between the implantable medical device and the external device, it is helpful to use a circularly polarized (CP) antenna, as it is independent of the orientation of the transmitter. An ISM 2.45 GHz band circularly polarized microstrip patch antenna for a conventional pacemaker was reported in^26^. But a CP antenna has not been proposed for the leadless pacemaker system. The other requirement of an implantable antenna is minimum detuning. For this point of view, a loop antenna is a good option. Loop antenna has small electric field and high magnetic field as compared to an electric type of antenna in the near field^27^ and is less susceptible to detuning.

This paper presents; a compact conformal loop antenna for the Medtronic Micra’s Transcatheter Pacing (TCP) system. The proposed antenna is a Hilbert curve inspired loop antenna with compact footprints (6 $\times$ 6 $\times$ 0.254 mm^3^), dual-band (1.4 GHz and 2.45 GHz), and circularly polarized in the 2.45 GHz band, as shown in Table 1. The 1.4 GHz frequency band is the Wireless Medical Telemetry Services (WMTS) defined in the United States by the Federal Communications Commission (FCC) for transmission of data related to patient health (biotelemetry) as per ^28^. Another operating band of the antenna is the 2.45 GHz ISM band, which can transfer the data at a high rate. The antenna was integrated with electronic IC models and tightly packed in the device enclosure; was evaluated by being placed at the center of the multi-layer canonical model of the human heart in a commercially available High-Frequency Structure Simulator (HFSS). To prove the efficacy of the proposed antenna in a realistic environment, we have put dummy electronics (IC and battery) in the capsule and surfaced the fabricated antenna around the inner wall of the 3D printed dummy capsule. Furthermore, the TCP system is experimentally validated in the ballistic gel phantom and then in the minced pork muscle.

The measured results were in good agreement with the simulated results. Ultimately, to obtain the compliance of the proposed antenna with human safety standards, SAR values for 1* g* human tissue as per FCC and 10* g* human tissue as per IEC values are evaluated and lie in the recommended range. We have analyzed the link budget numerically to calculate the telemetry range of the implantable antenna.

We have organized the manuscript in the following sections: “Methodology” Section explains antenna design of the system, modeling in HFSS, and optimization of antenna design parameters. “Results and discussion” section explains measurement set-ups for reflection coefficient (|S_11_|), axial ratio (A.R), gain, and comparison of results (simulated and measured). “SAR analysis” and “Link budget analysis” section discussed SAR and link budget. “Conclusion” section provides the conclusion of this research.

## Methodology

### System’s design

Research on deeply implantable antennas proposed for a leadless pacemaker is quite limited because it is challenging to design implantable antennas due to the limited space, multipath losses caused by lossy human tissues and the surrounding electronics modules. In this paper, the leadless pacemaker’s capsule diameter and length are as per Medtronic’s MICRA TCP system^13^. We also tried to mimic the outer and inner environment of the TCP system to design the proposed implantable antenna. We have used ceramic alumina (biocompatible material) capsule of a diameter of 6.7 mm and a length of 26 mm. The outer body of the TCP system includes: an electrode, fixation tines, anode ring, and lid but the pulse generator & sensor module, telemetry module, antenna, control & processing module, and battery are inside the capsule’s body. The perfect electrical conductor (PEC) material selected for the electrode, fixation tines, anode ring, and the battery and the modules are of Rogers RT/Duroid 6010 LM.

### Simulation setup for the proposed antenna

**Figure 3:**
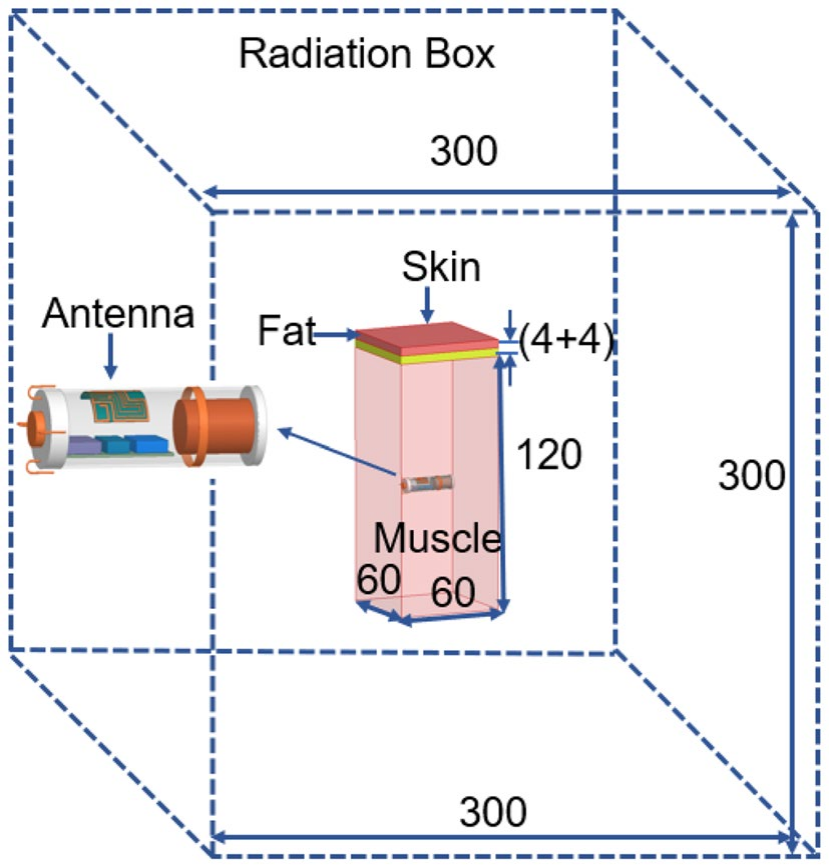
Simulation set-up for the proposed antenna in a multi-layer phantom model for the leadless transcatheter pacing system.

The in-vivo channel exhibits different characteristics than the outside body channel. The EM wave which propagates inside the body is significantly affected by the scatterers in the near field region. The use of analytical methods needs simplifications to characterize the in-vivo wireless communication channel. On the other hand, numerical methods are less complicated and suitable for approximations to the Maxwell’s equations through different techniques, like the Finite-Difference Time-Domain (FDTD), Uniform Theory of Diffraction (UTD), Finite Element Method (FEM), and Method of Moments (MoM)^29^. In this paper, the Finite Element Method (FEM) based commercially available ANSYS High-Frequency Structure Simulator (HFSS) characterizes the in vivo wireless communication channel. The design of the antenna took place inside the leadless pacemaker body (with dummy modules and the battery) and was subsequently implanted in a muscle layer at the center of a 60 mm × 60 mm × 128(4 + 4 + 120) mm multi-layer (skin + fat + muscle) phantom model, as shown in Fig. 3. The radiation boundaries are taken at a distance greater than *λo*/4 away from the antenna at 1.4 GHz. The electrical properties of the phantom model are frequency-dependent, and the proposed antenna must work in 1.4 GHz and 2.45 GHz ISM bands. So, to design a phantom model, the average value of the dielectric properties of corresponding bands is taken as per^30^. The electrical properties of skin, fat, and muscle at 1.4 GHz and 2.45 GHz, referenced from^23,31,32^ have been given in Table 2.

**Table 1:** Comparison of the proposed antenna with the other reported antennas.

Ref	^18^	^20^	^21^	^22^	^23^	Proposed
Antenna type	Conformal	Conformal	Flat	Flat	Flat	Conformal
Area (mm^2^)	22.5 × 30	17.28 × 14.28	Π × 5^2^	7 × 6.5	3 × 4	6 × 6
Volume (mm^3^)	22.5 × 30 × 0.05	17.28 × 14.28 × 0.28	Π × 5^2^ × 0.89	7 × 6.5 × 0.377	3 × 4 × 0.5	6 × 6 × 0.254
Freq (GH_z_)	0.402	2.45	0.402	0.402 1.6 2.45	2.45	1.4 2.45
Ground plane	Slotted	Full	Slotted	Slotted	Full	Full
Shorting pin	Yes	No	No	Yes	No	No
Implant depth	$-$	$-$	$-$	$-$	60 mm	64 mm
Impedance B.W (%)	3.73	$-$	$-$	36.8 10.6 8.9	21.8	17.1 12.8
A.R.B.W (%)	$-$	$-$	$-$	$-$	$-$	$-$ 11.42
Peak gain (dBi)	− 32	− 35	$-$	− 30.5 − 22.6 − 18.2	-25.9	− 32.7 − 25.92
SAR (W/Kg) 1 g/10 g	513.7/136.4	$-$	$-$	588/92.7 441/85.3 305/81.8	270.2/31	256.9/29.1 142.6/21.3

**Table 2:** Electrical properties of biological tissues^23,31,32^.

Tissue	1.4 GHz		2.45 GHz	
	${{\varvec{\epsilon}}}_{{\varvec{r}}}$	**σ**	${{\varvec{\epsilon}}}_{{\varvec{r}}}$	**σ**
Skin	39.66	1.036	37.88	1.44
Fat	5.39	0.065	5.28	0.10
Muscle	54.11	1.14	54.81	2.25

### Optimization of the transcatheter pacemaker system’s antenna

**Figure 4:**
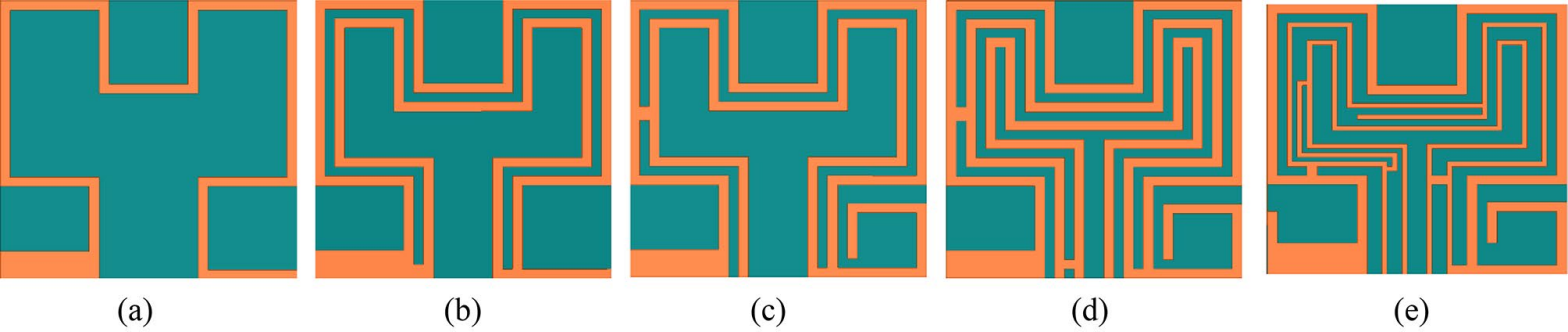
Antenna’s design evolution (**a**) step-I (**b**) step-II (**c**) step-III (**d**) step-IV (**e**) proposed antenna (step-V).

**Figure 5:**
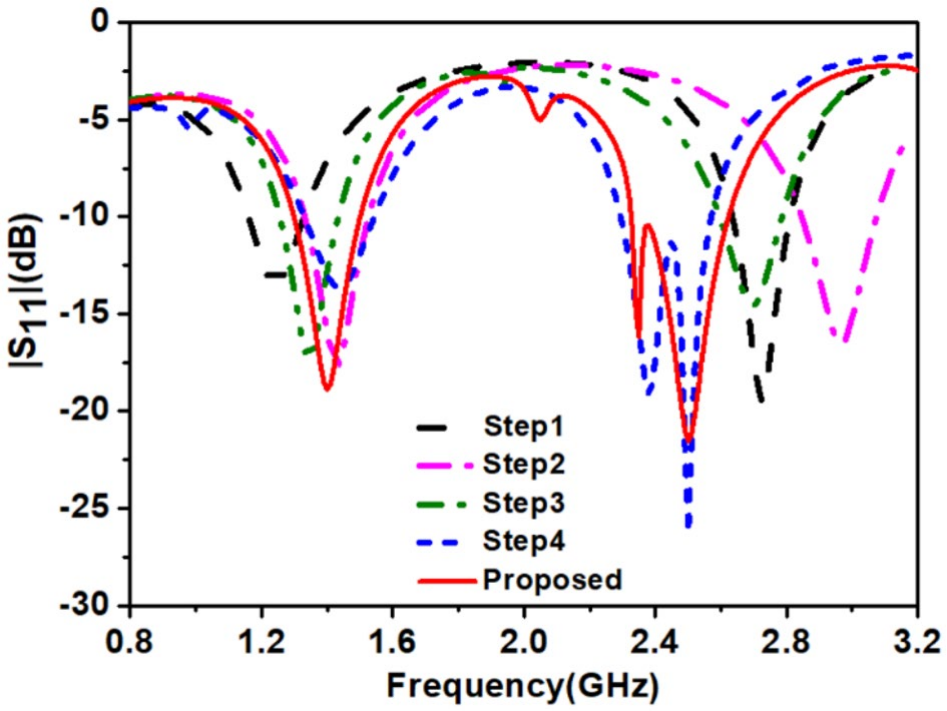
|S_11_| of optimization steps.

We have explained the optimization of the proposed antenna in five steps shown in Fig. 4a–e and Fig. 5 show the corresponding simulated reflection coefficients (|S_11_|). At first, we designed a planar Hilbert open loop antenna, Fig. 4a. The width of the loop is 0.2 mm, but the feeding arm thickness was 0.6 mm to get good impedance matching. The antenna designed in step 1 resonated at 1.24 GHz and 2.72 GHz. At 1.24 GHz, impedance matching was poor, and at 2.72 GHz, impedance matching was good, and their corresponding bandwidths are 160 MHz (1165.0–1325.0) and 175 MHz (2635.0–2810), respectively. At the lower resonance, the current path length was *λg*/6, and at the upper resonance path length of the current was *λg*/4. In step 2, second loop (*loop-2*) was created inside the first loop (*loop-1*) and shorted to loop-1 at both ends by shorting strips (*SS*_*1*_ and *SS*_*2*_), Fig. 4b. At this stage, the antenna was a closed-loop antenna, and it was resonating at 1.4 GHz and 2.9 GHz frequency bands with improved impedance bandwidths, 180 MHz, and 224 MHz, respectively. The current flowing into two paths: through *loop-1* and *loop-2* in the same direction, it increased impedance bandwidth but shifted the frequency bands to the higher side. But the higher band needs tuning to the 2.45 GHz band. In step 3, the shorting strip *SS*_*1*_. shifted upward and, an inverted U-shaped asymmetric stub (*Stub-1*) was connected, which increased the current path and shifted the frequencies to 1.35 GHz and 2.7 GHz.

**Figure 6:**
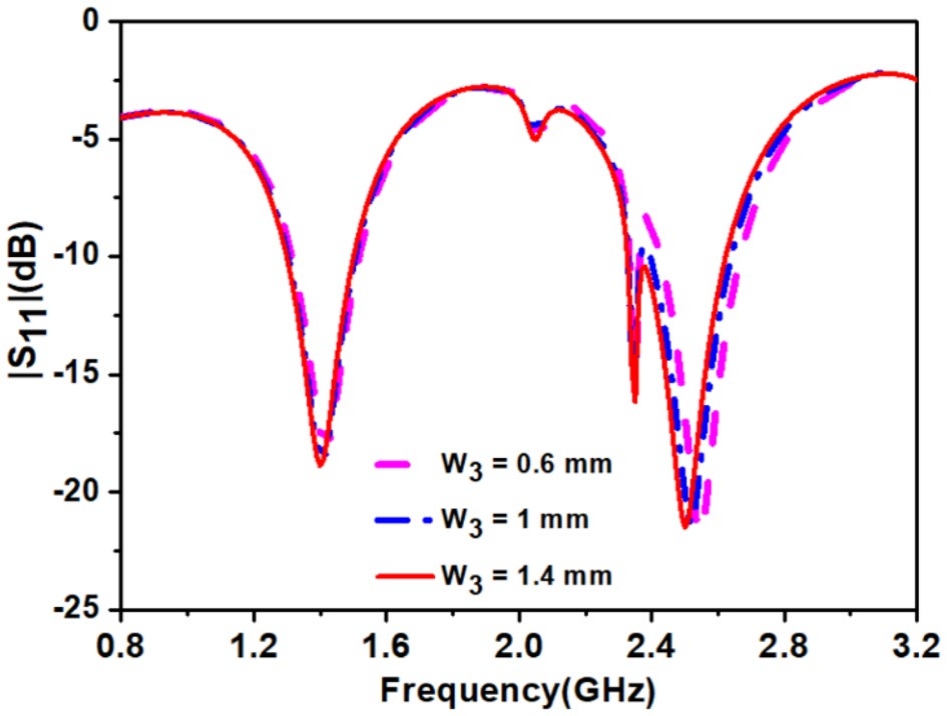
Effect of width (*w*_*3*_) of *stub-1* on |S_11_|.

If width(*w*_*3*_) of the inverted U-shaped slot is less than 1 mm it reduces the bandwidth at 2.45 GHz band as shown in Fig. 6. In step 4, the shifting of 2.7 GHz to 2.45 GHz was obtained by connecting loop-3 to loop-2 by *SS*_*3*_*.* In this way, due to interconnection between loop-2 and loop-3, two resonant frequencies at 2.34 GHz and 2.48 GHz were obtained and merged, created an improved bandwidth of 270 MHz (2280–2550). At 1.4 GHz band, improved impedance bandwidth of 207 MHz (1333–1540) was obtained, but impedance matching was not good.

**Figure 7:**
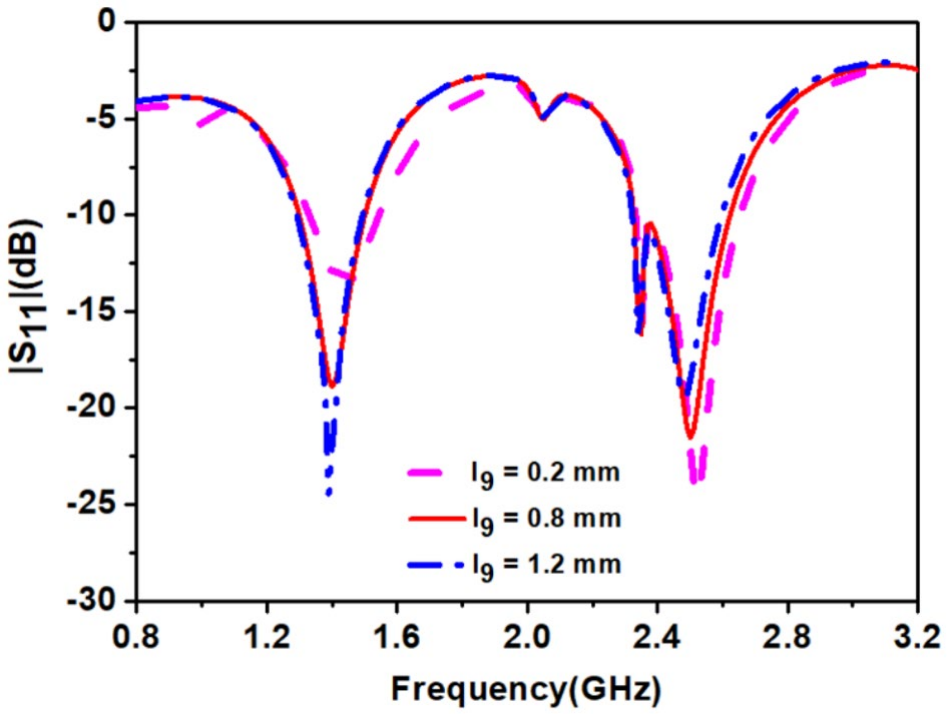
Effect of length (*l*_*9*_) of *stub-*2 on |S_11_|.

**Figure 8:**
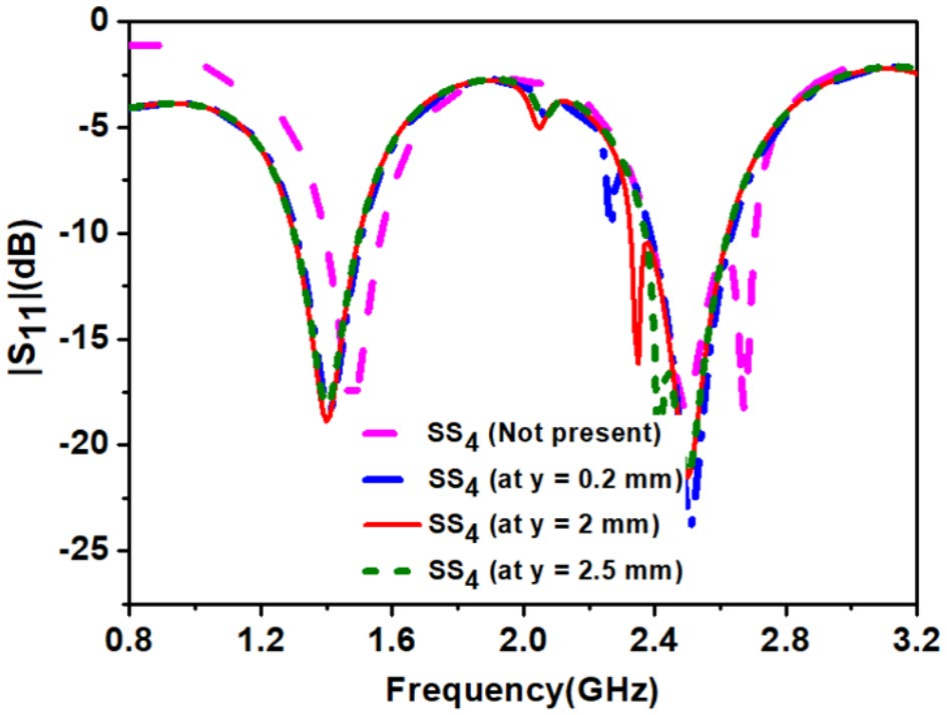
Effect of shorting strip (*SS*_*4*_*)* on |S_11_|.

In the final step, we connected *Stub-2* to the antenna. The length of the *Stub-2* controls the impedance matching at 1.4 GHz as shown in Fig. 7, and width controls the bandwidth, and we optimized results at 0.8 mm length and 0.2 mm width. The circular polarization (discussed in the next section) at the 2.45 GHz band was obtained by *Stub-3*, but it shifted the 1.4 GHz resonance higher side. The length of the *SS*_*4*_ (shorting strip) increased after creating the space between *loop-2* and *loop-3*. The optimization of length of *SS*_*4*_ is shown in Fig. 8, which re-tuned lower resonance to 1.4 GHz. The effect of width reduction of the loops on the antenna's performance was negligible. Hence, *Stub-2*, *Stub-3,* and *SS*_*4*_ provide good impedance matching and improved impedance bandwidth at 1.4 GHz and 2.45 GHz frequency bands.

### Dual-band circularly polarized implantable antenna

**Figure 9:**
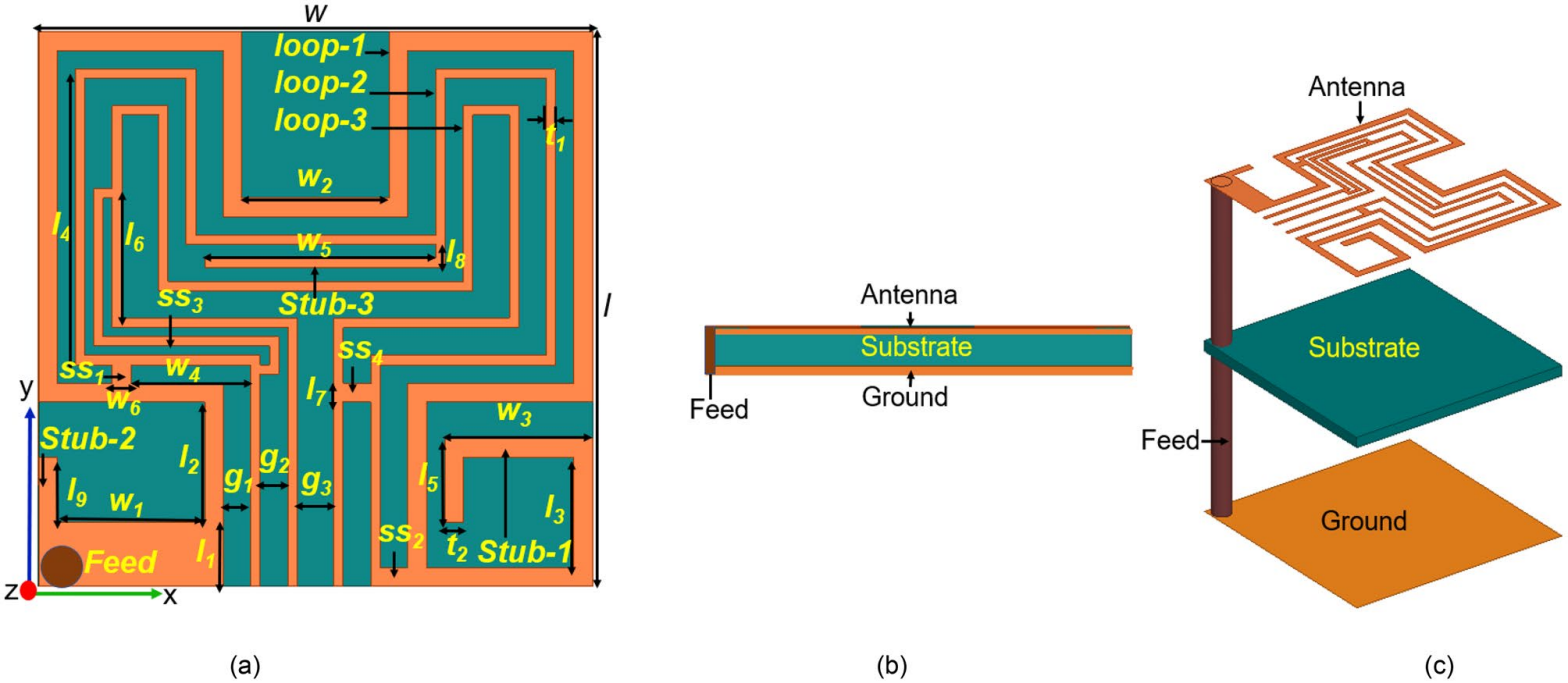
Geometry of the proposed dual-band antenna for the leadless cardiac pacemaker. (**a**) Front view (Loop patch) (**b**) Side view (**c**) Exploded view.

Figure 9 shows the complete design of the proposed antenna: Fig. 9a shows the front view of copper loop antenna, and Fig. 9b and c shows the side view and exploded view of the antenna.

The footprints of the proposed antenna are 6 × 6 × 0.254 mm^3^, and the dimensions have been given in Table 3. While designing an antenna for capsule devices, we must consider, space available for all the components inside the capsule, the coupling of antenna fields to the circuit and batteries, and the detuning of antenna bands because of the heterogeneous environment. In this context, we have proposed a dual-band circularly polarized conformal loop antenna. This antenna is designed on RT/Duroid 6010 LM with dielectric constant, ${\epsilon }_{r}$=10 and a thickness of 0.254 mm. The higher value of the dielectric constant helps in miniaturization. The substrate is flexible and very thin that can wrap around the inner body of the capsule without cracking hazards. The loop antenna design follows the Hilbert curve. The proposed antenna includes three Hilbert loops: *loop-2* fits inside the empty area of *loop-1*, and *loop-3* fits inside *loop-2*. The interconnections between two loops are optimized to get good impedance matching, bandwidth, and circular polarization. The connected three loops of antenna increases the overall electrical length hence, provides miniaturization along with the multi-band behavior^33^. The antenna has a complete ground plane that guarantees the radiation in the broadside direction of patch with desired high front-to-back ratio i.e., outwards from the body towards the external monitoring device.

**Table 3:** Design parameters of the proposed antenna (in mm).

Symbol	Value (mm)	Symbol	Value (mm)	Symbol	Value (mm)
*l*	6	*l*_*8*_	0.25	*w*_*6*_	0.2
*l*_*1*_	0.7	*l*_*9*_	0.8	*g*_*1*_	0.3
*l*_*2*_	1.6	*w*	6	*g*_*2*_	0.3
*l*_*3*_	1.4	*w*_*1*_	2	*g*_*3*_	0.4
*l*_*4*_	3.2	*w*_*2*_	1.6	*t*_*1*_	0.1
*l*_*5*_	0.7	*w*_*3*_	1.4	*t*_*2*_	0.2
*l*_*6*_	1.5	*w*_*4*_	1.5	$-$	$-$
*l*_*7*_	0.2	*w*_*5*_	2.5	$-$	$-$

**Table 4:** Maximum SAR and maximum allowable input power.

Frequency	SAR(W/Kg) (Input power = 1 W		Max. allowable input power (mW)	
	1* g*	10 g	1 g	10 g
1.4 GHz	256.9	29.1	6.22	68.7
2.45 GHz	142.6	21.3	11.2	93.8

**Table 5:** Link budget parameters.

Transmitter		
	Frequency (GHz)	1.4/2.45
*G*_*t*_	Antenna gain (dBi)	− 32.7/− 25.9
*P*_*t*_	Transmitter power (dBm)	− 16
	EIRP (dBW)	− 48.7/− 41.9
**Receiver**		
*G*_*r*_	Receiver antenna gain (dBi)	2.15
*T*_*o*_	Ambient temperature (K)	293
	Boltzmann constant	− 1.38E−23
*N*_*o*_	Noise power density (dB/Hz)	− 203.9
**Signal quality**		
*B*_*r*_	Bit rate (Mbps)	1
*E*_*b*_*/N*_*o*_	Ideal PSK (dB)	9.6
*G*_*c*_	Coding gain(dB)	0
*G*_*d*_	Fixing deterioration (dB)	2.5

Figure 9b and c show the side and exploded view of the proposed antenna, respectively. The design of the antenna acquired its final shape in the presence of a battery and other modules such as pulse generator, sensor, control & processing module inside the ceramic alumina capsule. The dimensions of the capsule’s body are as per Micra’s TCP system because this approach makes the antenna more appropriate for a realistic environment.

### Operation of the antenna for circular polarization

**Figure 10:**
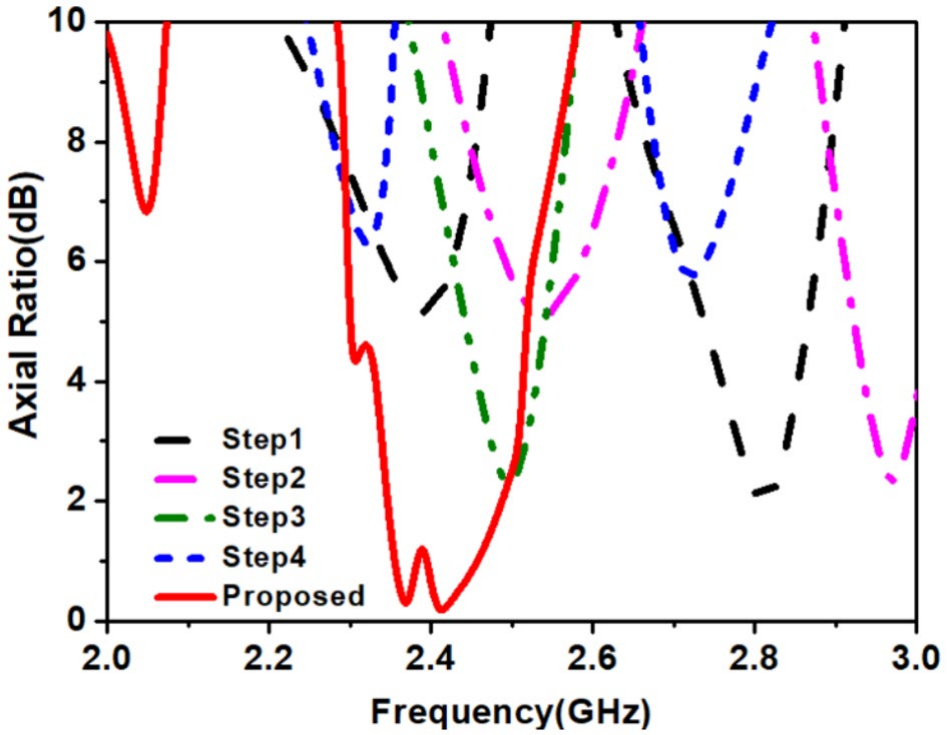
Simulated axial-ratio plots of optimization steps.

This section explains the circular polarization (CP) mechanism at the 2.45 GHz band of the proposed antenna. The antenna radiates CP radiation when it remains conformal to the inner wall of a TCP’s body, implanted in a muscle layer of the multilayer tissue model. Figure 10 shows the simulated plots of axial-ratio (A.R) for all the evolution steps. In step 4, when *loop-3* was connected, both (1.4 GHz and 2.45 GHz) the bands tuned to the desired frequency range, but it made ARBW out of the desired band, as shown in Fig. 10.

**Figure 11:**
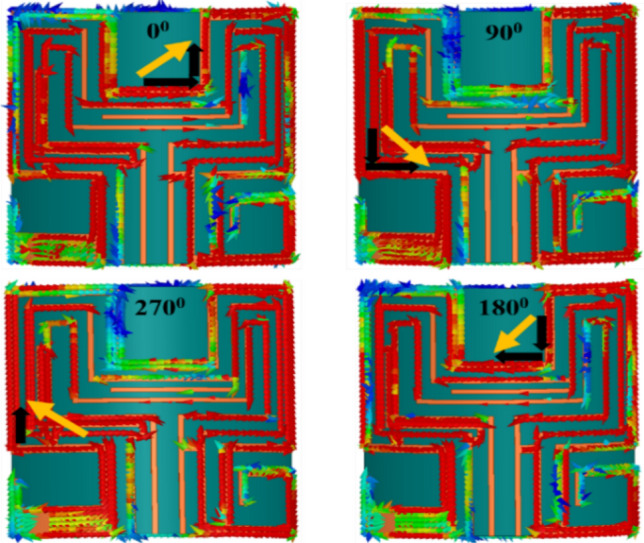
Surface current distribution on the proposed antenna at 2.45 GHz for *ωt* = 0°, 90°, 180° and 270°.

**Figure 12:**
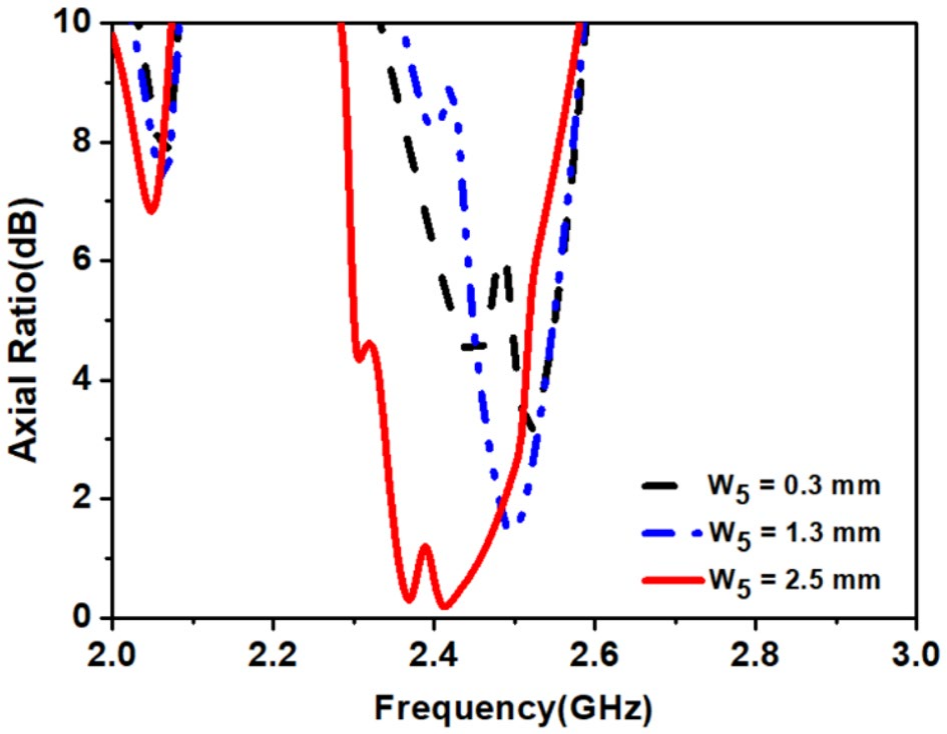
Effect of width (*w*_*5*_) of *Stub-3* on axial- ratio.

**Figure 13:**
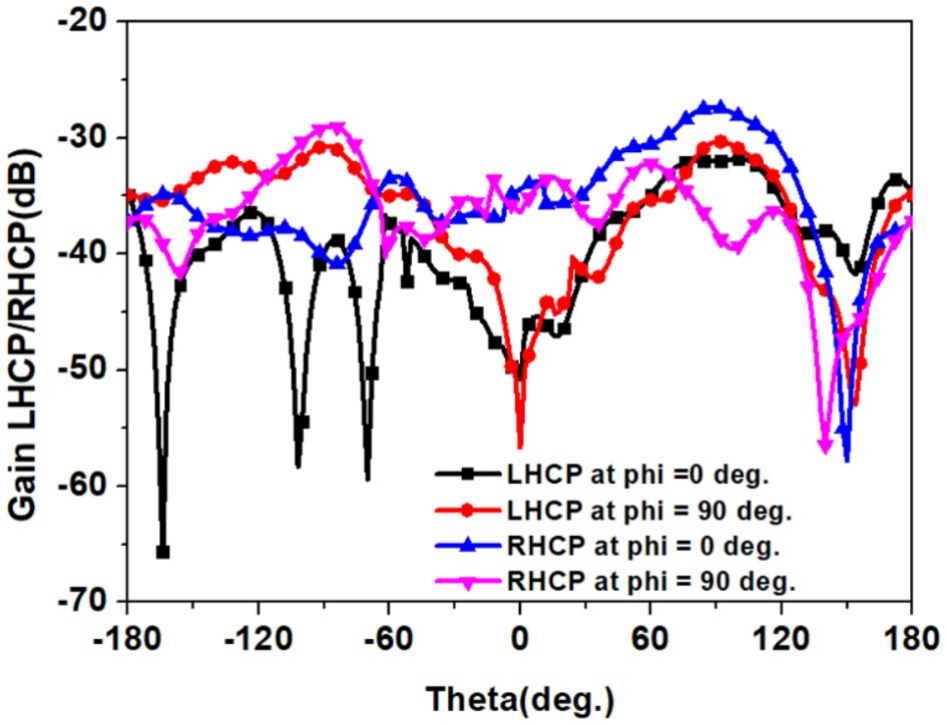
Simulated *co-* and $\times$-polarized gain patterns of the proposed integrated antenna at 2.45 GHz.

In the final step, we connected the L-shape stub (*Stub-3*) to loop-1. The *Stub-3* incorporated phase quadrature in the resultant current vector, observed at time phase *ωt* = 0° and 90° so, we obtained A.R < 3 dB in the 2.4 GHz band. The *Stub-3* rotates the current vector in an anticlockwise direction from the reference feed point in the left corner, as shown in Fig. 11. We also noticed that if the width (*w*_*5*_) of *Stub-3* increased, ARBW widened but shifted to the lower side of the frequency band, as shown in Fig. 12. Figure 13 shows simulated gain patterns for the proposed antenna at 2.45 GHz. It is evident from the figure that the *co*-polarized right-hand circular polarization (RHCP) is 20 dB more than the $\times$-polarized left-hand circular polarization (LHCP), which is sufficient to produce *co*-polarized waves.

### Evaluation of antenna performance in heterogeneous environment

The performance analysis of the integrated antenna in an anatomical tissue model is necessary that checks the antenna behavior for a realistic human body environment. Anatomical models are produced based on high-resolution images of actual individuals. This study uses, AustinMan v 2.6 anatomical model, developed by the National Library of Medicine’s VHP (Visible Human Project) data^34^. There are a total of 104,328,722 voxels in the AustinMan v 2.6 model, and each voxel has a resolution of 1 × 1 × 1  mm^3^. Noted, bounding box of dimensions 400 × 400 × 400 mm^3^ represent the section of the torso model used in simulation.

**Figure 14:**
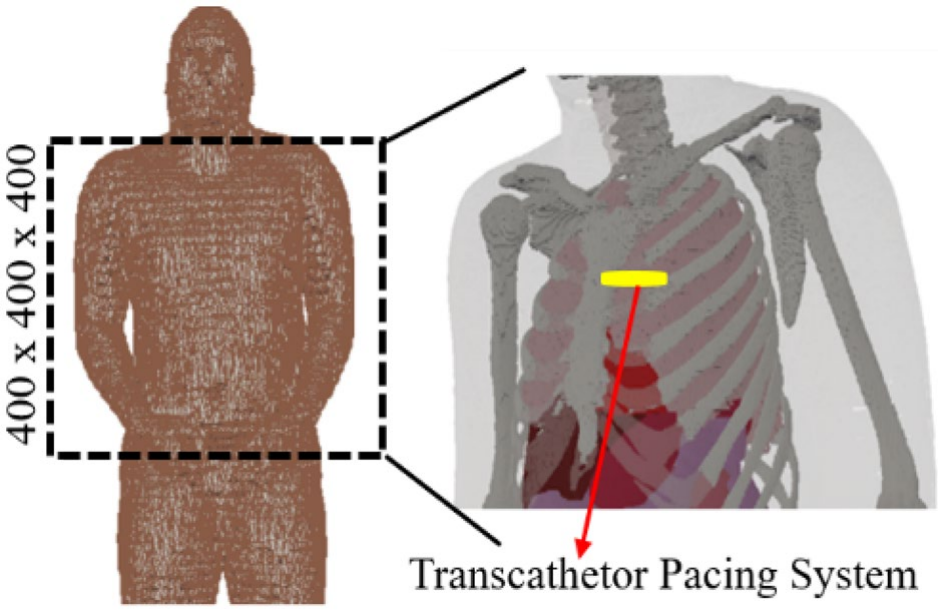
TCP’s capsule location inside heart’s muscle of the AustinMan model.

**Figure 15:**
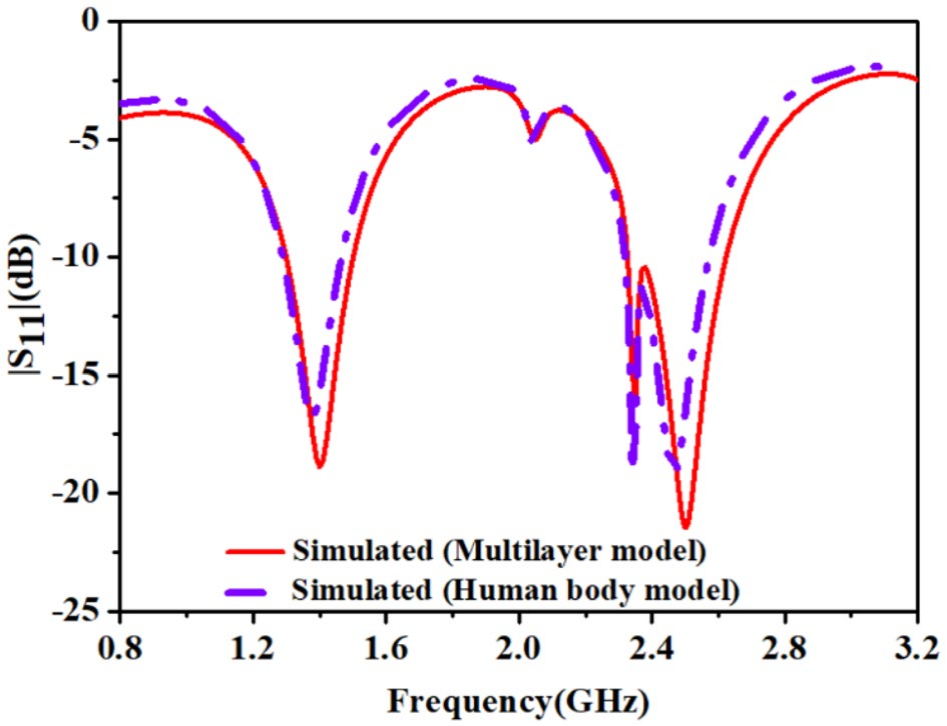
Comparison of simulated |S_11_| in multi-layer homogeneous model and heterogeneous human body model.

**Figure 16:**
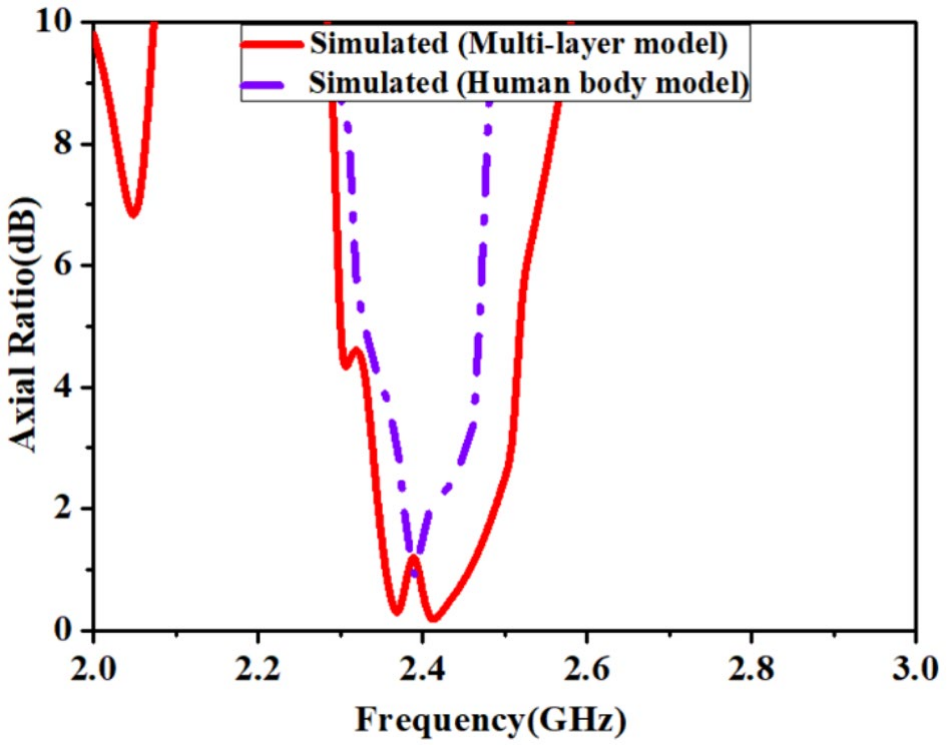
Comparison of simulated A.R in multi-layer homogeneous model and heterogeneous human body model.

We have analyzed the antenna inside the heart muscle of a high-fidelity anatomical voxel model (AustinMan v 2.6) in CST Microwave studio, as shown in Fig. 14. AustinMan v2.6 is a heterogeneous anatomical body model. Therefor the permittivity of the heart muscle has different values for all four chambers of the heart, the leadless pacemaker’s capsule is surrounded by the different primitivities (${\epsilon }_{r}$= (50–56)). Due to the heterogeneity of the heart, the high permittivity surrounding tissues resulted shift of the resonant frequencies to a little lower side, as shown in Fig. 15. At 1.4 GHz, the resonant frequency shifts to 1.38 GHz but is less affected due to the low value of conductivity. But resonance at 2.5 GHz shifts to 2.46 GHz due to relatively high conductivity of tissues. The impedance bandwidth and axial ratio bandwidth, as shown in Figs. 15 and 16 respectively but wide enough to cover the two reported bands (1.4 GHz and 2.45 GHz).

## Results and discussion

### Near-field measurement

**Figure 17:**
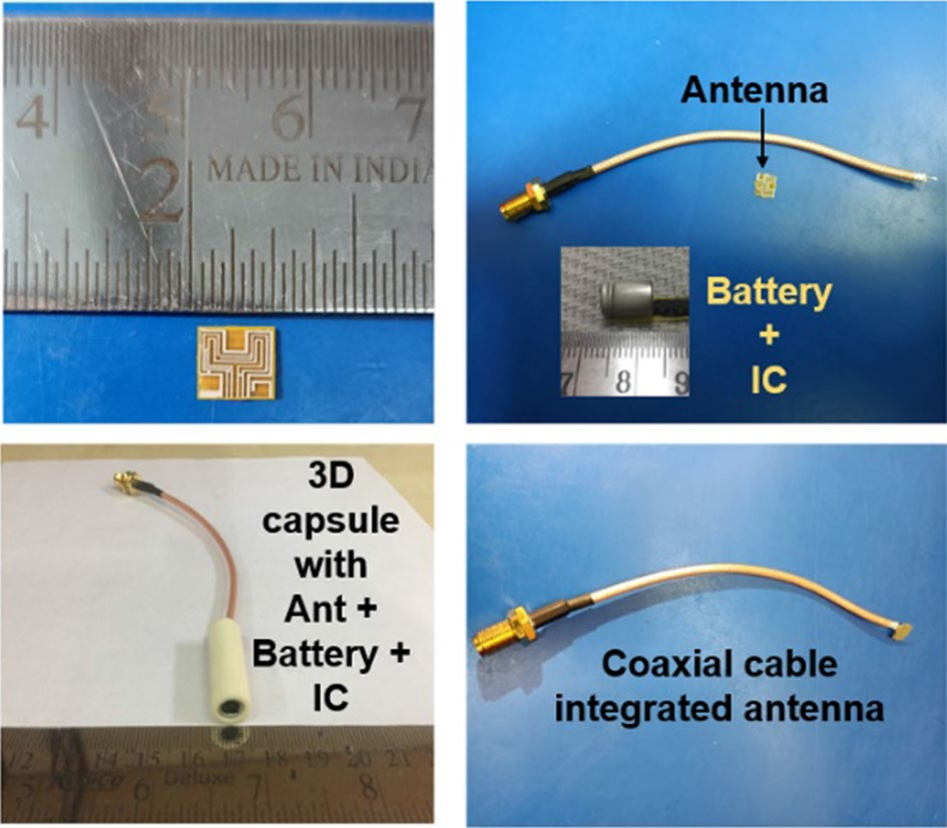
Fabricated prototype of the antenna and 3D printed ceramic alumina capsule with cable integrated antenna and dummy electronics.

**Figure 18:**
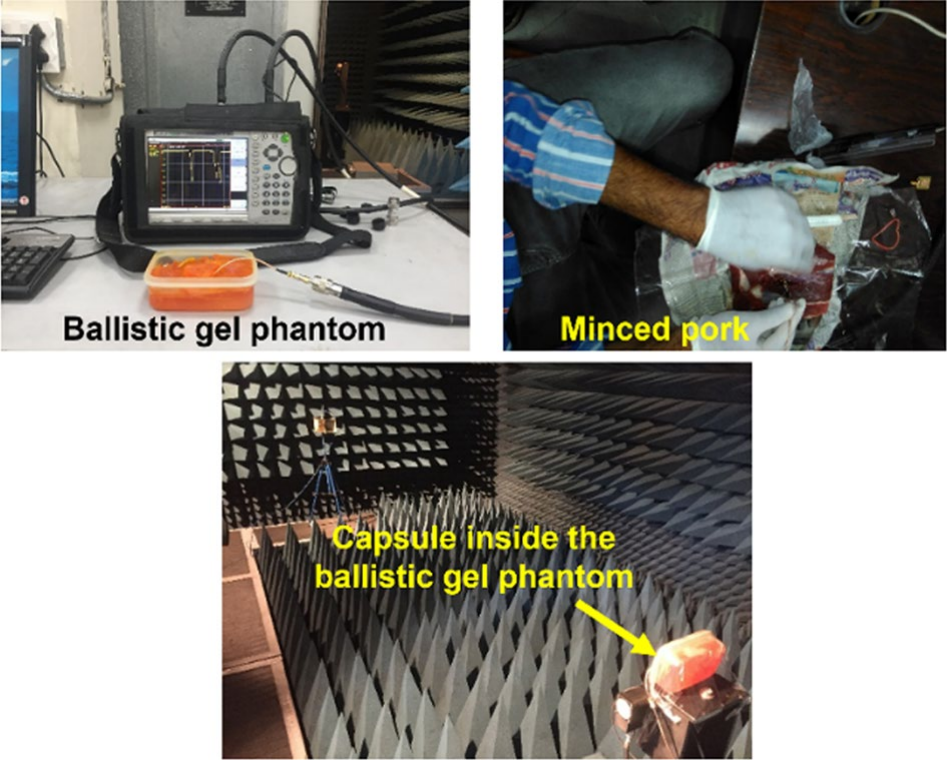
Near-field and far-field measurement set-up for the proposed integrated antenna in open lab environment and anechoic chamber, respectively.

Figure 17 shows the fabricated capsule prototype: an antenna, dummy battery, and IC placed inside the 3D printed ceramic alumina hollow cylinder. The performance of an integrated antenna system is measured in the vicinity of the ballistic gel phantom^35^ and minced pork, respectively, as shown in Fig. 18.

**Figure 19:**
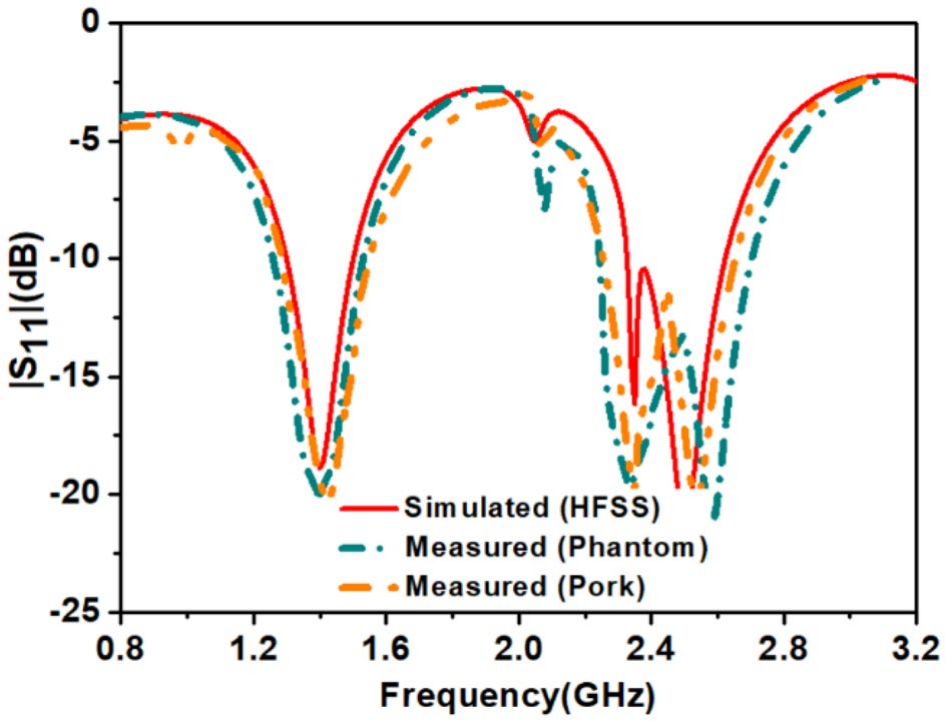
Comparison of simulated and measured (|S_11_|).

The dielectric property of the minced pork depends on the many factors such as: it’s freshness, water holding capacity, and temperature. In our case, pork was at room temperature and its permittivity was 54.5 and due to less water content (σ = 1.2), bandwidths at both the reported bands widened as shown in Fig. 19. The phantom that we have used is ballistic gel and it’s recipe is explained in ^35^. The measured values of permittivity and conductivity of the ballistic gel are 54.1 and 0.9 respectively. Therefore, due to low conductivity of the ballistic gel phantom the bandwidths at the reported bands are widened. The permittivity of the pork was measured using open ended coaxial probe method. The open-ended coaxial probe is the most used method to measure the dielectric properties of tissues/phantoms due to its simplicity. The sample handling is non-destructive for both ex vivo and in vivo measurements. However, the open-ended coaxial method assumes a homogeneous sample that is in good contact with the probe; therefore, air bubbles and uneven sample surfaces may lead to slight differences^36^. The coaxial cable is polythene shielded to prevent direct contact with phantom and minced pork in the measurement. This arrangement could also minimize, the otherwise undesirable field coupling to the cable^37,38^. Figure 19 shows the comparison of simulated and measured near-field parameter, reflection coefficient (|S_11_|) of an integrated antenna system. In both the medium: ballistic gel phantom, and pork, measured results cover efficiently reported bands. Some discrepancies were observed after the implantation of the prototype into the pork in the measured results. The cause of the difference was the airgaps between the tissues, which creates path losses. In minced pork, impedance bandwidth at 1.4 GHz and 2.45 GHz are 250 MHz and 430 MHz, respectively. In the ballistic gel phantom, the measured results closely follow the simulated one because of the closeness of the properties to the heart muscles. In the phantom, impedance bandwidth at 1.4 GHz and 2.45 GHz are 410 MHz and 520 MHz, respectively. As the antenna size is smaller than the coaxial cable, therefore, it is also essential to discuss the behavior of the cable that is connected to the antenna and, it affects the performance of the antenna and it shift the lower resonant frequencies to the lower side of the frequency band^39^. In the simulation environment, we have taken 1 mm of cable length. But in the real-time leadless pacemaker: antennas, telemetry modules, and other modules share one board with an integrated power supply, so cable effects are not present. The measured results in both environments: ballistic gel phantom and minced pork, were achieved as anticipated.

### Far-field measurement

**Figure 20:**
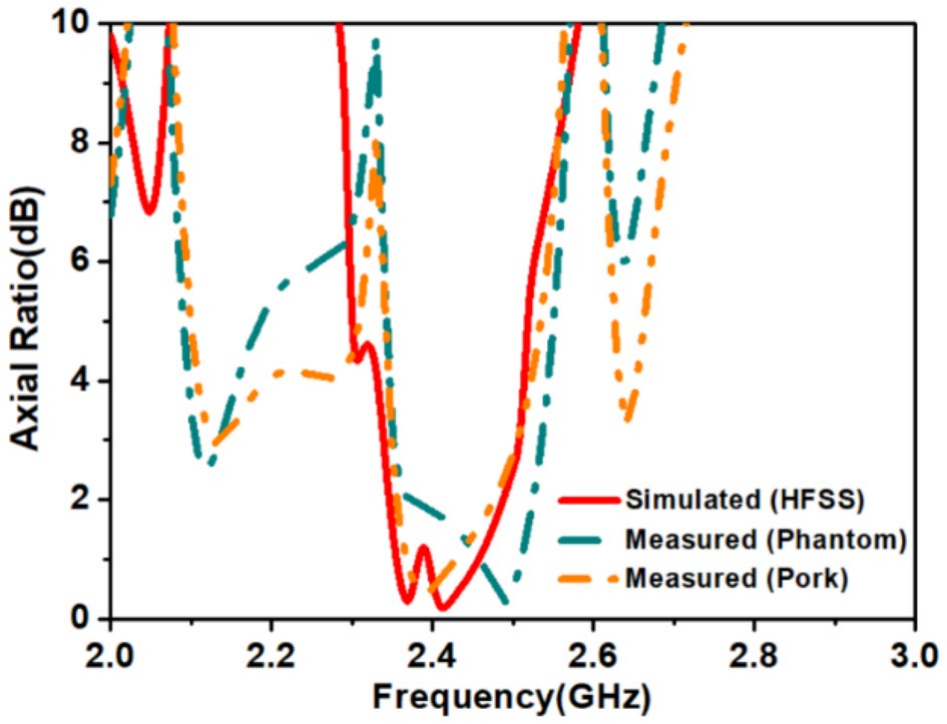
Comparison of simulated and measured axial-ratio.

**Figure 21:**
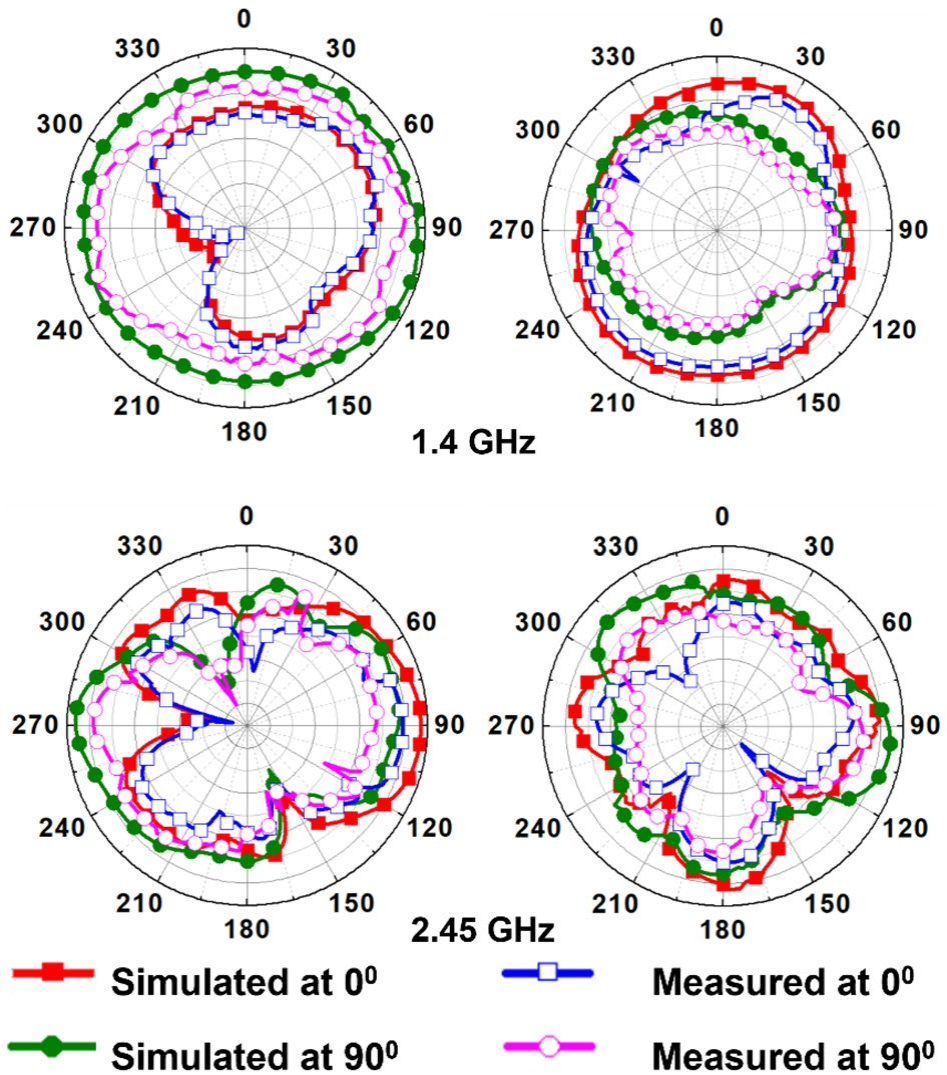
Comparison of simulated and measured radiation patterns of the proposed integrated antenna in pork for: (**a**) *E*-plane (*ϕ* = 90°) (**b**) *H*-plane (*ϕ* = 0°).

The circularly polarized omnidirectional antenna can mitigate communication losses due to misalignment between the implanted antenna and the outside antenna of the patient monitoring device. Figures 20 and 21 show the comparison between simulated and measured axial-ratio and the radiation pattern, respectively. The simulated ARBW is 180 MHz. The proposed prototype: antenna and electronics integrated inside the capsule, was tested in the ballistic gel phantom in an anechoic chamber to measure the far-field parameters, axial-ratio, and radiation patterns in *E*-plane (*ϕ* = 90°) and *H*-plane (*ϕ* = 0°), as shown in Fig. 18. The antenna exhibited a nearly omnidirectional gain pattern at both 1.4 GHz and 2.45 GHz. The measured peak gain of the proposed antenna was − 33.2 dBi at 1.4 GHz and − 28.5 dBi at 2.45 GHz whereas, simulated peak gain at 1.4 GHz and 2.45 GHz bands were − 32.7 dBi and − 25.9 dBi, respectively.

## Communication Link

### SAR analysis

**Figure 22:**
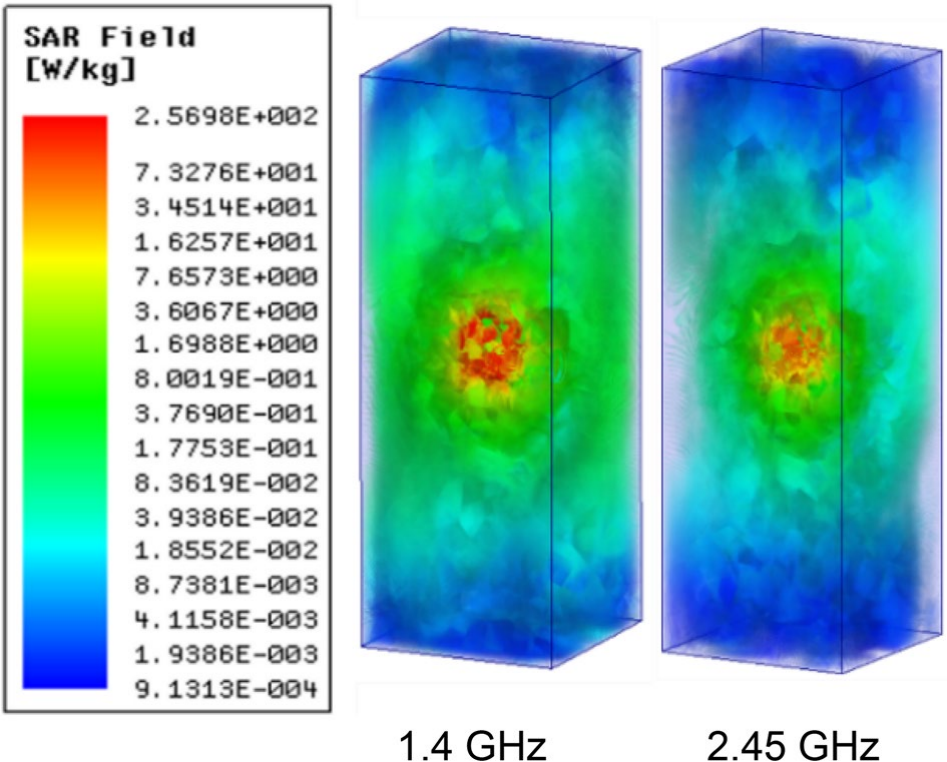
Simulated average SAR distribution caused by the proposed antenna over the 1* g* of tissue of heart’s multi-layer model.

**Figure 23:**
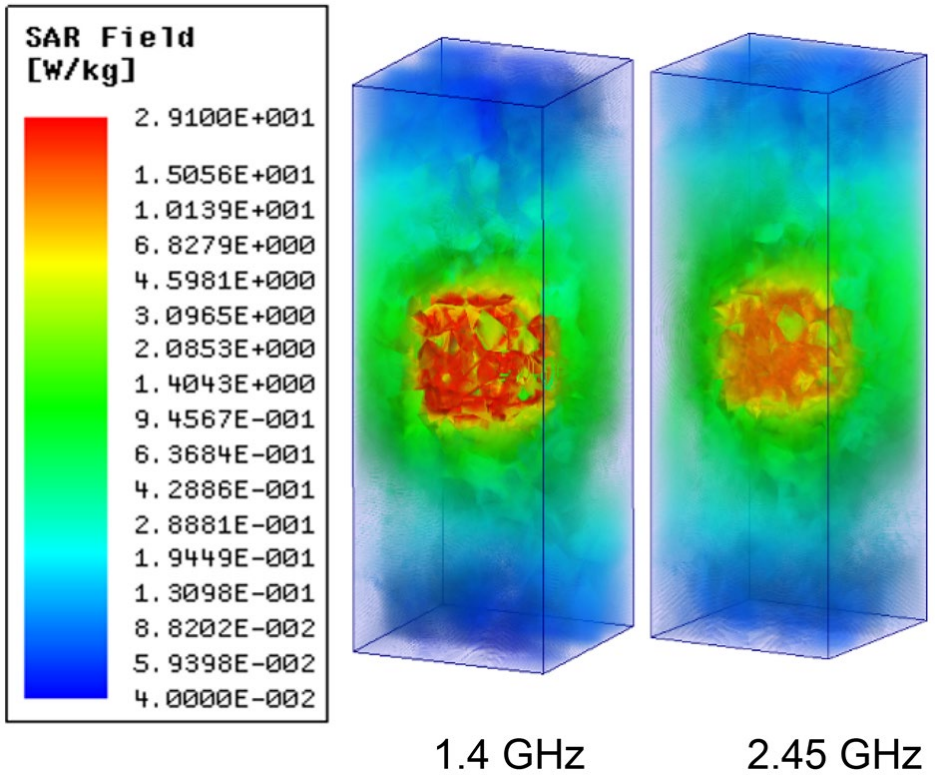
Simulated average SAR distribution caused by the proposed antenna over the 10* g* of tissue of heart’s multi-layer model.

We have evaluated SAR (Specific Absorption Rate) when the assembled prototype resides in the multi-layer phantom model, as shown in Figs. 22 and 23.

Following safety standards are considered: EMF, New ICNIRP Guidelines, and IEEE C95.1–2019 Standard IEEE^40^. Following the safety standards Averaged SAR (ASAR) value for 1* g*/10* g* of human tissue in the form of a cube must be not more than 1.6/2.0 W/kg. Table 4 shows the 1* g*/10* g* ASAR values for the proposed antenna system for 1 W input power. The ASAR value decreases gradually with increasing frequency, and surface current with high intensity covers larger apertures at low frequency leading to a high value of ASAR.

Whereas for higher frequency bands, the comparatively smaller radiating aperture is having high-intensity current distribution leading to a low ASAR value. For the highest 1* g*/10* g* ASAR value of 256.9/29.1 W/Kg at 1.4 GHz, the corresponding maximum allowable input power is 6.22/68.7 mW, respectively. The input power restriction for IMDs is only 0.025 mW, so, based upon this investigation, the proposed system would not be life-threatening.

### Link budget analysis

For pacemakers such as the Micra TCP system, data from the implant is transmitted to the healthcare professional by an external patient monitoring device named “my care link”. So, to establish a stable link between the implant and external monitoring system to transmit real-time biological signals, the communication link budget is calculated. In order to avoid complexity, the link budget is calculated using Friis transmission equations, which includes transmit antenna gain (G_t_), receive antenna gain (Gr), the free space path loss (L_f_), and assuming Line of Sight (LOS) between the transmitter and receiver. So, to establish the communication between the transmitter and receiver, the link c/N_o_ (carrier to Noise density ratio) of the system must be more than the required c/N_o_ (Receiver’s sensitivity), as per^41,42^.

Hence, the following equations apply;

1 $$LM\left(dB\right)=link \frac{c}{{N}_{0}}\left(dB\right)-required\frac{c}{{N}_{0}}\left(dB\right)$$

2 $$required \frac{c}{{N}_{0}}\left(dB\right)={E}_{b}+10 {log}_{10}\left({B}_{r}\right)- {G}_{c}+ {G}_{d}$$

3 $$link \frac{c}{{N}_{0}}\left(dB\right)=EIRP-{L}_{f}+{G}_{r}-{N}_{0}$$

4 $$EIRP\left(dB\right)={P}_{t}+{G}_{t}$$

5 $${L}_{f}=20{log}_{10}\left(\frac{4\pi x}{\lambda }\right)$$

where *P*_*t*_ is the transmit power, *G*_*t*_ and *G*_*r*_ are the gain of the transmitting and receiving antennas, respectively, *E*_*b*_ is the energy per bit and *B*_*r*_ is the bit rate, *N*_*o*_ is the noise power density, and *L*_*f*_ is the free space path loss with distance *x* between the transmitting and receiving antenna. At the transmitting and receiving end, we ignored (for simplicity) polarization mismatch losses and impedance mismatch losses.

**Figure 24:**
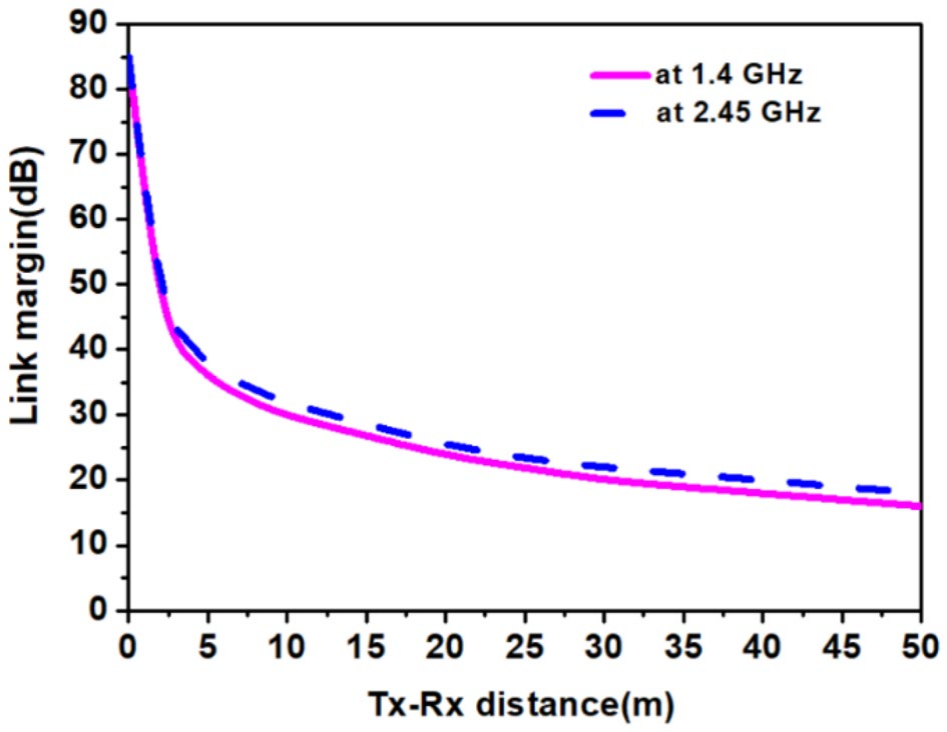
Link margin between *Tx* and *Rx* antennas at both the reported bands with a data rate of 1Mbps.

Figure 24 shows a graph between the link margin and telemetry distance (*T*_*x*_–*R*_*x*_ distance), calculated using Table 5. In link margin calculations, the proposed antenna; was assumed transmitting antenna, and the monopole antenna (gain 2.15 dBi) was a receiving antenna. From the graph, it can be seen that the difference between the link margin at 1.4 GHz and 2.45 GHz with respect to the telemetry distance is very less with minor deviation. Usually, both link margin and telemetry distance reduce due to the high path losses at the higher frequency for similar gain values at both higher and lower frequencies. But, in our case the reduction due to the higher path loss at 2.45 GHz is compensated with the higher gain value at 2.45 GHz in contrast to the value at 1.4 GHz. The similar trend is also noticeable in^43^. Hence, for the bit rate of 1 Mbps, the link margin was more than 16 dB for 50 m of distance in the 1.4 GHz and 2.45 GHz frequency band, which is good enough for practical applications^44^.

## Conclusion

This paper proposes a compact dual-band antenna for a leadless cardiac transcatheter pacing system. The proposed antenna is in conformal shape inside the body of a biocompatible ceramic alumina capsule with the complete ground plane, which avoids the possibility of tissue interaction with the surrounding electronics. The sufficient bandwidths and gain in both the reported bands: 1.4 GHz and 2.45 GHz, were obtained. We measured S-parameters and radiation patterns of the antenna with capsule in ballistic gel phantom and minced pork. The simulated and measured results agreed in the desired frequency bands. The SAR analysis for 1* g* and 10* g* tissues was done, as per New ICNIRP Guidelines and IEEE C95.1–2019 Standard IEEE C95. We found that the designed antenna can handle more than the necessary power (25 µW) for pacemaker applications. Additionally, to define the wireless communication ability of the antenna, the link margin has been calculated at 1 Mbps data rate. This antenna is appropriate for modern-day’s Micra TCP and Nanostim LCP leadless cardiac pacing systems, owing to its compact size, conformal shape, dual-band behavior, gain, and circular polarization.
